# Prospective Randomized Controlled Pilot Study of High-Intensity Lightbox Phototherapy to Prevent ICU-Acquired Delirium Incidence

**DOI:** 10.7759/cureus.14246

**Published:** 2021-04-01

**Authors:** Kermit S Zhang, Tomer Pelleg, Shahzad Hussain, Venkateswara Kollipara, Anthony Loschner, Mahtab B Foroozesh, Edmundo Rubio, Frank Biscardi, Susanti R Ie

**Affiliations:** 1 Pulmonary, Critical Care and Sleep Medicine Section, Department of Medicine, Virginia Tech Carilion School of Medicine, Roanoke, USA; 2 Critical Care Medicine, Samaritan Medical Center, Portland, USA

**Keywords:** icu delirium, bright light therapy, quality improvement and patient safety, circadian rhythm, prolonged mechanical ventilation, delirium in icu

## Abstract

Background

This study aimed to evaluate the role of disturbed circadian rhythm in potentiating intensive care unit (ICU)-acquired delirium.Previous studies have demonstrated bright light therapy (BLT) as an effective modality to improve sleeping patterns and cognitive function in non-critically ill patients. However, its benefit in the ICU has not been clearly established. In this study, we aimed to evaluate the application of daily high-intensity phototherapy at the bedside to deter ICU delirium incidence and duration.

Methodology

This was a single center, prospective study conducted in ICUs at the Carilion Roanoke Memorial Hospital in Roanoke, VA. Adults patients admitted to the ICU from July 9, 2018 to March 20, 2020 were included in the study. The patients were subjected to 30-minute BLT session (10,000 lux) at the bedside starting at 0700 while in the ICU. Patients were randomized into either the control group (standard hospital lighting) or phototherapy group. Data were analyzed using Wilcoxon rank sum test for continuous variables, Pearson chi-square test for categorical variables, and logistic regression for multivariable analysis that examined significant risk factors for ICU delirium.

Results

Delirium incidence between BLT (18%) and control (17.5%) groups was non-significant. Total number of delirium-free, coma-free days, as determined by Confusion Assessment Method for the ICU, demonstrated no differences between groups with a median of 28 days (p = 0.516). In multivariable analysis, patients with a Sequential Organ Failure Assessment Score >3 also showed no significant change in ICU delirium incidence when provided bedside BLT compared to those with standard hospital lighting (odds ratio: 0.08; 95% confidence interval: 0.002-1.40; p = 0.867).

Conclusions

In this randomized control pilot study, daily morning 10,000 lux BLT of 30-minute duration alone was not associated with a significant decrease in ICU-acquired delirium incidence or duration compared to standard hospital lighting. Future studies should consider a nuanced approach to better elucidate the role of disturbed circadian rhythm in influencing ICU-acquired delirium by not only undertaking BLT during the day but also minimizing nighttime light exposure.

## Introduction

Delirium is a non-specific but reversible acute change in baseline mentation that can arise from many causes that cannot be accounted for by a baseline neurocognitive disorder or a severely reduced level or arousal. The Diagnostic and Statistical Manual of Mental Disorders, fifth edition defines delirium as one that encompasses a change in consciousness, cognition, and perception accompanied with an acute onset and transient fluctuation throughout the day. It is a serious condition that has been associated with poor clinical outcomes, including increased days on mechanical ventilation, length of hospital stay, long-term cognitive impairment, cost of care, and mortality. Although a delirium diagnosis characterizes a patient’s current cognitive state, it does not identify an etiology, thus prompting further investigation associated with the hospital course or current illness. Treatments common with antipsychotics have limited efficacy and risk for significant side effects including extra-pyramidal and cardiac effects [1,2].

It has been reported that delirium can occur in 16 to 89% of hospitalized patients with the highest incidence in the intensive care unit (ICU), with up to 80% of mechanically ventilated patients having delirium [3]. However, diagnosis in the ICU is challenging, with as many as 75% of delirium cases going undiagnosed without the routine and regular use of validated screening tools [4]. Importantly, a patient must be arousable to assess for delirium which is assessed by an agitation/sedation tool such as the Richmond Agitation-Sedation Scale (RASS) [5]. The Confusion Assessment Method for the ICU (CAM-ICU) and the Intensive Care Care Delirium Screening Checklist are the most widely studied and commonly used delirium screening tools [3]. Delirium risk factors are divided into two categories: predisposing risk factors reflective of a patient’s medical history, and precipitating risk factors based on the healthcare setting. Among the latter, alterations in circadian rhythm and sleep disorders are frequently observed among the critically ill [1]. In addition to mechanical ventilation, sedatives, and disease severity, the lack of natural light exposure in the ICU may play an essential role as light is the most powerful cue for the circadian rhythm [6]. Several studies have shown that bright light therapy (BLT) can realign disturbed circadian rhythm in non-critically ill populations, such as among the geriatric population living in nursing homes [7,8]. Its efficacy has also been reported in mild disorders, such as jet leg disorders, and psychiatric disorders, such as seasonal affective disorder. As delirium is associated with disrupted circadian rhythm and sleep, BLT may be a useful resource to reduce its incidence or severity.

A follow-up study of similar design by Simons et al. incorporated continuous 700 lux ceiling-mounted BLT in the ICU throughout the day for seven hours to assess its efficacy in deterring ICU delirium compared to control groups without BLT [9]. No significant differences were detected using the CAM-ICU in the number of delirium-free and coma-free days (DFCFD), ICU length of stay (LOS), or hospital LOS. Additional assessment of melatonin and cortisol excretion also showed no significant changes between groups. While these studies have shown to be either inconclusive or ineffective in deterring ICU delirium, trial designs in relation to our understanding of ICU light exposure and circadian rhythm warrant further investigation of ICU BLT application.

Although Simons et al. failed to find an effect on ICU delirium incidence, the maximal light intensity achieved for the intervention was 700 lux, which was substantially lower than average daylight. For comparison, sunlight may be as intense as 100,000 lux and even on a heavily overcast day is approximately 1,000 lux. Recent measurement of daytime light intensity across the ICU was low in comparison to natural daylight at approximately 150 lux, which is between 10- and 1,000 times dimmer than daylight [10]. In addition, a light intensity of 1,000 lux has been shown to be minimally necessary in maintaining circadian rhythms in healthy adults [11]. As a result, the trial may not have reached a sufficient light intensity to observe appropriate biological effect. Moreover, it has been shown that for BLT to be effective in maintaining circadian rhythm, light therapy need not be continuous. Past studies have demonstrated that exposure to three consecutive bright light pulses for a minimum of 15 minutes is more effective than continuous BLT [12]. This can not only be more practical for ICU application but also be more cost-effective for hospitals to incorporate. Therefore, we tested the hypothesis that the application of morning BLT for 30 minutes daily at 10,000 lux will be associated with patient improvement of DFCFD in the ICU.

A previous meta-analysis evaluated the efficacy of non-pharmacological interventions compared to standard of care on delirium incidence and duration in ICU patients from 15 trials [13]. No significant results were found to support the use of non-pharmacological interventions, including BLT. However, these findings contrasted sharply with the meta-analysis conducted by Shen et al. who reported that BLT reduces ICU-acquired delirium incidence when the Simons trial was excluded due to deficient light stimulation [14]. Altogether, this underscores that further investigations with statistically powered studies at substantiated therapeutic levels of intervention must be conducted before a reasonable conclusion on the efficacy of BLT on ICU-acquired delirium can be determined.

## Materials and methods

Study design

The BLT to Prevent ICU-Acquired Delirium trial is a single-center, randomized, prospective, open-label controlled trial conducted in the ICUs of the Carilion Roanoke Memorial Hospital (CRMH) in Roanoke, Virginia. In 2016, our documented incidence of delirium at CRMH was 20% among admitted ICU patients. Both medical and surgical ICUs were made available for the study. All ICU rooms had windows with daylight access primarily facing south and east. The study was approved by the Carilion Clinic Institutional Review Board.

Patient enrollment

All patients who were admitted between July 9 ,2018 and March 20, 2020 to the designated medical and surgical ICUs at CRMH were screened for eligibility. We enrolled 108 ICU patients for our study, but only 78 patients were able to complete the protocol as outlined. Patients had to be 18 years or older with an expected ICU stay of at least 24 hours. The eligible timeframe for patient enrollment into our study was within 24 hours of their ICU admission. This ensured investigators appropriate time for randomization and allowed the investigators to start patients on BLT if they were assigned to the treatment group. All patients or their power of attorney/next of kin provided written informed consent. We excluded patients with a confirmed psychiatric history of bipolar disorder due to the concern that BLT has been associated with inducing manic episodes.

Cohort randomization and masking

Patients were randomly assigned to BLT intervention or no BLT intervention (control) in a 1:1 ratio according to a predetermined computer-generated randomization list. Patients were enrolled by ICU physicians, residents, and medical students who worked on the study team. Masking of treatment allocation was not possible.

Bright light therapy application system

The bright lighting system used was a Carex Day-Light Classic Plus Bright Light Therapy Sun Lamp. It has a maximum intensity of 10,000 lux of glare-free white light and was placed by the patient’s bedside if randomized to the BLT treatment group. There was only one setting for the lamp, and each lamp unit was placed at a fixed distance that was between 12 to 18 inches from the head of the bed to maintain uniform light intensity exposure. A General Electrics’ 24-hour Plug-In Mechanical Timer was used for a self-timed BLT treatment session for 30 minutes every morning starting at 0700 and stopping promptly at 0730.

Intervention

Patients assigned to the BLT group were exposed to peaks of 10,000 lux white light starting at 0700. This light intensity was maintained until 0730 when the self-timer turned off the therapy lamp. Patients assigned to the BLT group after 0700 during randomization began BLT the following morning. Otherwise, patients were exposed to standard lighting settings of 150 lux in the ICU. Patients in the control group were only exposed to standard light settings of 150 lux. No serious adverse effects from BLT was expected, but patients were warned about possibilities of headache and/or photophobia. The BLT treatment continued daily for as long as they remained in the ICU. The patient’s ICU nurse was also aware of the scheduled time for the therapy at trial enrollment and informed a member of the investigative team if the patient was not present during their scheduled BLT session.

Assessment of delirium

Within 12 hours of a patient’s enrollment into the trial, patients were assessed for delirium risk by first measuring their level of consciousness using RASS. Patients who were able to demonstrate arousable levels of consciousness (RASS: -3 and above) were then assessed for delirium using the CAM-ICU. Assessment by both the RASS and CAM-ICU scores were used at least twice per day by different providers as part of the standard of care and was documented in the electronic medical record by the ICU care team.

Cumulative incidence of delirium was defined as the presence of delirium from at least one positive CAM-ICU (score of >3) during their ICU stay. The number of DFCFD in 28 days was calculated by subtracting the number of days patients had delirium or were comatose (RASS: -4 and -5) from 28. DFCFD were defined as when the RASS score was greater than -3 and the CAM-ICU score was negative for two consecutive assessments within 24 hours. If a patient was free from delirium for 48 hours, as demonstrated with two negative CAM-ICUs, the delirium was determined to be resolved. If patients were arousable but the CAM-ICU could not be scored, presence of delirium was determined by review of medical and nursing records for haloperidol use and delirium symptoms.

Outcome measures

The primary outcome measures were the cumulative incidence and duration of delirium as measured by DFCFD in 28 days. Secondary outcome measures included ICU admission sequential organ failure assessment (SOFA) score, duration of mechanical ventilation, sedative use (haloperidol, midazolam, dexmedetomidine), ICU LOS, and hospital LOS. Mortality throughout the hospital stay was also tracked. All measures were extracted from the patient’s EMR.

Statistical analyses

Analysis of outcome measures was performed by intention to treat (all randomized patients) and per protocol (patients exposed to the assigned lighting condition for at least 80% of the admission period and who completed the study without protocol deviation). As DFCFD represents a non-normal continuous variable, Wilcoxon rank sum test was chosen for statistical analysis. However, the Wilcoxon rank sum test is approximately 5% less power-efficient than the Student’s t-test; hence, a power analysis conducted for the Student's t-test was adjusted to compensate for this loss of efficiency. In an effort to detect a one-day difference in DFCFD, with the assumption that the average delirium-free duration is eight days with the standard deviation being two days, a total sample size of 100 patients was determined to achieve a power of 80% statistical power with two-sided α value of 0.05.

Subpopulation analyses were conducted by statistical modeling to evaluate the effects of BLT on delirium incidence in patient subgroups based on relevant ICU admission criteria and management. All statistical analyses were performed using R version 3.6.1 (R Core Team 2019) and the KernSmooth (v.2.23-15) packages. Continuous data were compared with the Wilcoxon rank sum test for non-normally distributed variables, the Pearson chi-square test was used for categorical variables, and logistic regression for multivariable analysis that examined significant risk factors for ICU delirium.

## Results

**Figure 1:**
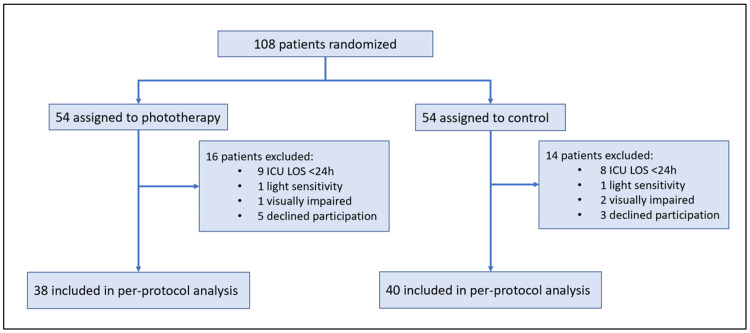
Schematic of randomized patient population enrolled in the pilot study. ICU = intensive care unit; LOS = length of stay

**Figure 2:**
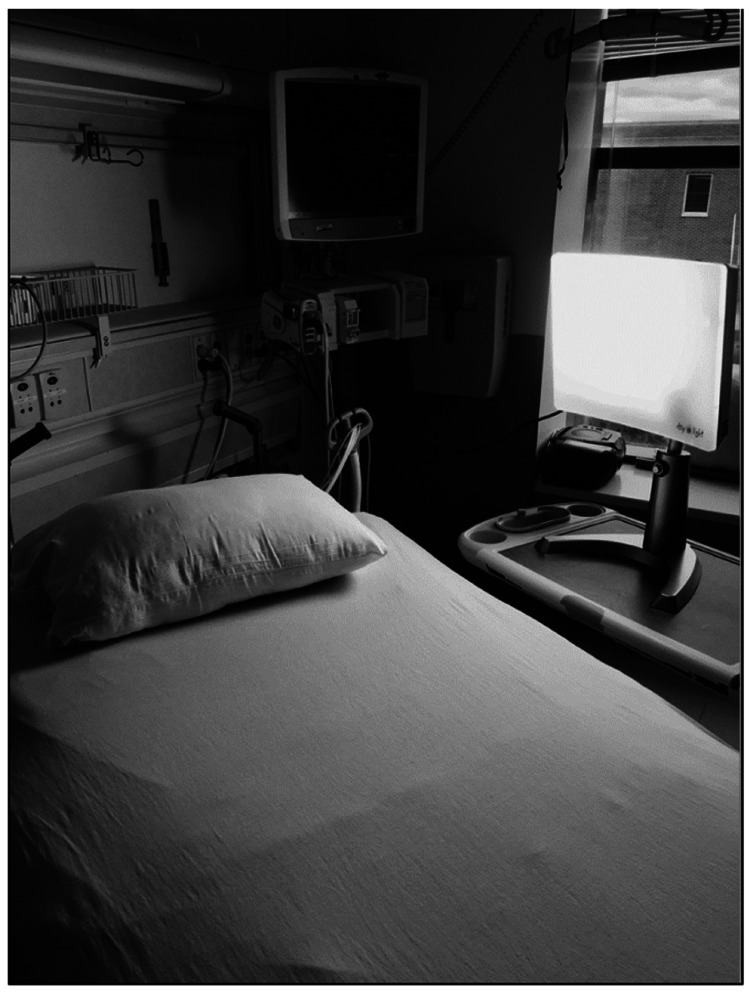
BLT ICU setup. BLT = bright light therapy; ICU = intensive care unit 10,000 lux light box setup for patients randomized to BLT group. Lights were placed at bedside near the head of the patient’s bed to maintain therapeutic distance. A plug-in mechanical timer switch (not shown) was used for a treatment session for 30 minutes every morning between 0700 and 0730. The ICU care team and the research group would check daily to ensure therapeutic distance and BLT was consistent throughout the patient’s ICU stay.

Between June 22, 2018 to March 19, 2020, a total of 108 patients were enrolled in the study. Among those enrolled and randomized, a total of 17 patients could not complete the study due to an ICU LOS of less than 24 hours and eight patients declined participation (Figure 1). The remaining 78 patients completed the study protocol as outlined. All participants had access to natural daylight in their ICU rooms. BLT patients were started on their light therapy sessions within 24 hours after ICU admission. Nurses assigned to these patients were given the contact information of the investigator currently working on the study, and were asked to notify the investigator of any potential patients not present in their rooms or any malfunctioning light boxes leading to missed therapy sessions. Adherence was 97.4% with one patient having a documented request to turn the light off 10 minutes into their scheduled 30-minute therapy session on their second to last day in the ICU. Patients receiving BLT were monitored daily by both ICU nurses and a member of the research team for changes in mental status associated with their clinical course, and to ensure that the lightbox which emitted 10,000 lux of light was maintained at a therapeutic distance of 12 to 18 inches as stated in the manufacturer’s instructions (Figure 2). Overall, the BLT was well tolerated by patients. Clinical management of patients was maintained by the ICU team. No adverse events from either the patients or ICU care team were reported.

Baseline patient risk factors demonstrated no differences among sex, BMI, and season at time of ICU admission (Table 1). The median age of the treatment and control groups were similar at 63.5 and 64, respectively. The SOFA score was also similar between groups. There were significantly more trauma and surgical ICU patients in the control group compared to the BLT group (p = 0.014). In addition, known risk factors for delirium were analyzed between the two cohorts. These included a documented history of alcohol abuse, diabetes, and a history of neurological impairment, all of which were similar in the two groups.

**Table 1:** Baseline patient characteristics. ICU = intensive care unit; SOFA = Sequential Organ Failure Assessment; COPD = chronic obstructive pulmonary disease ICU admission data and relevant patient past medical history associated with increased risk of delirium episodes. Significant differences if p-value < 0.05. Data are represented as number (%) or median (interquartile range)

	Phototherapy treatment (n = 38)	No treatment (n = 40)	P-Value
Patient risk factors on ICU admission			
Sex, Male	21 (55.3%)	24 (60.0%)	0.874
Age (years)	63.5 (46.5-75)	64 (48-75)	0.369
Body mass index	26 (24-34)	28 (24-34)	0.711
SOFA score	3 (2-6)	3 (1-6)	0.619
Season at time of admission			
Winter	25 (66%)	24 (60.0%)	0.488
Spring	5 (13.2%)	4 (10.0%)	0.732
Summer	4 (10.5%)	6 (15.0%)	0.741
Autumn	4 (10.5%)	6 (15.0%)	0.525
Reason for ICU admission			
Medical	22 (57.9%)	19 (47.5%)	0.493
Trauma/Surgical	3 (7.9%)	11 (27.5%)	0.014
Neurological	5 (13.2%)	4 (10.0%)	0.668
Sepsis	8 (21.1%)	6 (15.0%)	0.370
Past medical history			
Documented history of alcohol abuse	1 (2.6%)	3 (7.5%)	0.617
Current smoker	7 (18.4%)	6 (15%)	0.765
COPD	5 (13.2%)	9 (22.5%)	0.384
Neurological or behavioral impairment	18 (47.4%)	22 (55%)	0.657
Cardiovascular disease	20 (52.6%)	25 (62.5%)	0.507
Diabetes	15 (39.5%)	17 (42.5%)	0.999
Chronic kidney disease	10 (26.3%)	8 (20.0%)	0.595

The overall delirium incidence between the treatment and control groups were at 18% and 17.5%, respectively (Table 2). The median numbers of DFCFD for both cohorts was 28. Among those with delirium, the time (days) to first delirium day and duration of the delirium episode were shown to be statistically non-significant between groups. Patients with BLT therapy typically had an average of 1.5 hospital days until their first delirium episode, which persisted for a mean of 16 hours, as assessed by the CAM-ICU. The control group had one hospital day until their first delirium episode lasting an average 30 hours until return of baseline mental status. The median ICU LOS and total hospital LOS was not statistically significant between cohorts. Overall, 86.8% of patients in the BLT group and 95% of patients in the control group were discharged alive. The hospital mortality rate was also statistically non-significant between cohorts with 8% and 5% among the treatment and control group, respectively.

**Table 2:** Clinical outcomes. ICU = intensive care unit; DFCFD = delirium-free and coma-free days; LOS = length of stay Clinical outcomes between the BLT and control group. The patients’ clinical course was tracked and analyzed through the EMR. Select sedatives documented are based on the Carilion Clinic ICU sedation and pain management protocols. Significant differences if p-value < 0.05. Data are represented as number (%) or median (interquartile range) *Only one patient in total was documented to have received quetiapine; significance between dosages could not be determined. **Two patients, both in the control group, were documented to have received haloperidol; significance between dosages could not be determined

	Phototherapy treatment (n = 38)	No treatment (n = 40)	P-Value
Delirium			
Incidence	7 (18%)	7 (17.5%)	0.999
Total number of coma or delirium days	0 (0–1)	0 (0–1)	0.516
Total number of DFCFD	28 (27–28)	28 (26–28)	0.516
Delirium duration (hours)	16 (10–38)	30 (18–56)	0.275
Time to first delirium day (days)	1.5 (1–3)	1 (0–2)	0.292
Clinical outcomes			
Number of patients discharged alive	33 (86.8%)	38 (95%)	0.362
Median ICU LOS (days)	3 (2–5.5)	3.5 (2–5)	0.939
Median hospital LOS (days)	6 (4–13)	6 (4–10)	0.592
ICU mortality	3 (8%)	2 (5%)	0.612
Hospital mortality	5 (13.2%)	2 (5%)	0.314
Sedative use			
Fentanyl			
Number of patients administered	11 (29%)	14 (35%)	0.258
Median micrograms/day	84.4	137.5	0.793
Dexmedetomidine			
Number of patients administered	5 (13%)	4 (10%)	0.732
Median micrograms/day	253.9 (234–352)	540 (36–1781)	0.999
Quetiapine			
Number of patients administered	1 (2.6%)	0	0.482
*Median micrograms/day	47.9	--	--
Midazolam			
Number of patients administered	6 (16%)	8 (20%)	0.551
Median milligrams/day	5.5 (2–47)	4 (2–6)	0.331
Haloperidol			
Number of patients administered	0	2 (5%)	0.494
**Median dose (mg)/day	--	3.5 (2–5)	--
Mechanical ventilation			
Mechanical ventilation in the ICU	11 (29%)	8 (20%)	0.800
Duration of mechanical ventilation (days)	6 (3–10)	3.5 (2–4)	0.125
Median number of self-extubations	0 (0–1)	0 (0–2)	0.311

With regards to ICU patient management, the number of patients requiring mechanical ventilation was non-significant between groups (Table 2). The average duration of mechanical ventilation for BLT patients was six days compared to 3.5 days in the control group. There were no self-extubations that occurred in either group. The percentage of patients given sedatives (fentanyl, dexmedetomidine, quetiapine, midazolam) throughout their ICU stay was found to be similar between groups as well. Haloperidol was administered to two patients at a median dose of 3.5 mg/day, both of whom were randomized to the control group. No patients receiving BLT were administered haloperidol. Analysis of patients who experienced at least one delirium episode in the hospital demonstrated they were more severely ill with a higher SOFA score compared to those who did not have delirium (Table 3). No significant differences were seen in regard to age, sex, BMI, or season at time of ICU admission between groups.

**Table 3:** Patient characteristics between delirium versus no-delirium patients. ICU = intensive care unit; SOFA = sequential organ failure assessment; COPD = chronic obstructive pulmonary disease ICU admission data and relevant patient past medical history based on patients who had or had not experienced a delirious episode. Significant differences if p-value < 0.05. Data are represented as number (%) or median (interquartile range)

	Delirium (n = 14)	No delirium (n = 64)	P-Value
Patient risk factors on ICU admission			
Age (years)	63.5 (51–71)	64 (46–77)	0.587
Sex, Male	9 (64.3%)	36 (56.3%)	0.860
SOFA score	6 (4–7)	3 (1–5)	0.003
Body mass index	29 (22–31)	28 (24–34)	0.710
Season at time of admission			
Winter	10 (71.4%)	39 (60.9%)	0.760
Spring	1 (7.1%)	8 (12.5%)	0.999
Summer	2 (14.3%)	8 (12.5%)	0.999
Autumn	1 (7.1%)	9 (14.1%)	0.679
Reason for ICU admission			
Medical	7 (50.0%)	34 (53.1%)	0.819
Trauma/Surgical	2 (14.3%)	12 (18.8%)	0.766
Neurological	3 (21.4%)	6 (9.4%)	0.204
Sepsis	2 (14.3%)	12 (18.8%)	0.448
Past medical history			
Documented history of alcohol abuse	2 (14.3%)	2 (3.1%)	0.136
Current smoker	3 (21.4%)	10 (15.6%)	0.689
COPD	2 (14.3%)	12 (18.8%)	0.999
Neurological or behavioral impairment	5 (35.7%)	35 (54.7%)	0.379
Cardiovascular disease	6 (42.9%)	39 (60.9%)	0.379
Diabetes	4 (28.6%)	28 (43.8%)	0.549
Chronic kidney disease	0	18 (28.1%)	0.032

Further comparison between patients who did and did not have at least one delirium episode demonstrated significant differences in clinical outcomes (Table 4). Patients with delirium had overall worse ICU and hospital mortality. They were also significantly more likely to be on mechanical ventilation compared to those who had not experienced delirium (p < 0.0001). The duration of mechanical ventilation and number of documented self-extubations remained similar between groups. Regarding sedation in the ICU, the frequency and dosage of sedatives for delirium patients were found to be significantly different. Overall, 92.9% of patients who had at least one episode of delirium were prescribed fentanyl at a median dose of 373 mcg/day compared to 51.6% of patients who did not experience any delirium and received a median dose of 100 mcg/day (Table 4). Similar high proportions of sedatives such as dexmedetomidine and midazolam were determined to be higher among delirium patients, but the median dosage/day were non-significant between groups.

**Table 4:** Clinical outcomes between delirium and no-delirium patients. ICU = intensive care unit; LOS = length of stay Clinical outcomes between patients who had or had not experienced delirium. Select sedatives documented are based on the Carilion Clinic ICU sedation and pain management protocols. Significant differences if p-value < 0.05. Data are represented as number (%) or median (interquartile range) *Only one patient in total was documented to have received quetiapine; significance between dosages could not be determined

	Delirium (n = 14)	No delirium (n = 64)	P-Value
Clinical outcomes			
Number of patients discharged alive	10 (71.4%)	61 (95.3%)	0.435
Median ICU LOS (days)	4 (3–11)	3 (2–5)	0.194
Median hospital LOS (days)	6 (4–16)	6 (4–11)	0.999
ICU mortality	4 (28.6%)	1 (1.6%)	0.003
Hospital mortality	4 (28.6%)	3 (4.7%)	0.044
Mechanical ventilation			
Mechanical ventilation in the ICU	9 (64.3%)	21 (32.8%)	<0.0001>
Duration of mechanical ventilation (days)	3 (2–10)	4 (3–5.5)	0.801
Number of self-extubations	0	0	0.173
Sedative use			
Fentanyl			
Number of patients administered	13 (92.9%)	33 (51.6%)	0.155
Median micrograms/day	373.3 (100–850)	100 (50–220)	0.045
Dexmedetomidine			
Number of patients administered	4 (28.6%)	3 (4.7%)	0.044
Median micrograms/day	506 (150–940)	216 (100–470)	0.730
Quetiapine			
Number of patients administered	1 (7.1%)	0	0.173
Median micrograms/day	47.9	0	--
Midazolam			
Number of patients administered	6 (42.9%)	4 (6.3%)	0.012
Median milligrams/day	4.4 (2–47)	4.8 (1–5)	0.560

**Figure 3:**
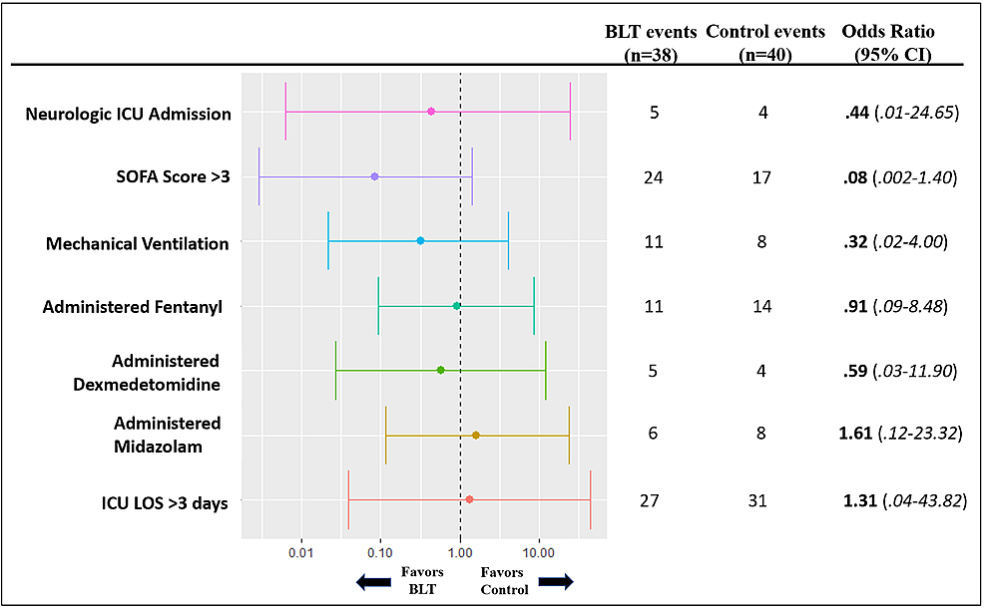
Effect of BLT on delirium incidence among risk factor subpopulations. ICU = intensive care unit; SOFA = sequential organ failure assessment; BLT = bright light therapy Forest plot of risk factor subpopulations among ICU patients comparing the effects of BLT on delirium incidence. Analyses were done based on statistical modeling of the risk factors found in Tables 3 and 4. Results are plotted on a logarithmic scale

Subgroup analysis was conducted to further evaluate the effects of BLT in the ICU with risk factors correlated to increased delirium risk (Figure 3). Among patients admitted to the ICU suspected of having an underlying neurologic etiology, the odds of developing delirium after randomization in the BLT treatment group was 0.44 times less likely compared to those not receiving BLT. However, the results were not statistically significant as the 95% confidence interval ranged between 0.01 and 24.65. Statistical modeling of six additional risk factors was also demonstrated to be statistically insignificant. The odds ratio (OR) of several of these factors were noted to be less than one, suggesting decreased odds of developing delirium among BLT patients compared to those in the non-BLT control group. Those factors included a SOFA score >3, requiring mechanical ventilation in the ICU, receiving fentanyl, and receiving dexmedetomidine with an OR of 0.08, 0.32, 0.91, and 0.59 respectively. Patients who were administered midazolam or had an ICU LOS more than three days had calculated odds ratio of 1.61 and 1.31, respectively, suggesting increased odds of developing delirium among BLT patients compared to that of the control group. However, none of these results were statistically significant.

## Discussion

In our study, BLT did not reduce the cumulative incidence of ICU delirium. The duration of delirium between the treatment and control groups demonstrated a non-significant trend toward shortened episodes for BLT patients. Secondary outcomes including hospital LOS, mortality, sedative use, and mechanical ventilation also showed no difference between groups. These preliminary findings aligned with the results documented by Simons et al., who found no differences in delirium incidence and duration among ICU patients given continuous, low-intensity light therapy [9]. This underscores that the disrupted circadian rhythm in ICU patients is impacted by additional compounding influences unique to the critical care environment, including noise from monitors and drug effects. As delirium is a multifactorial syndrome, observational studies have demonstrated that as many as 25 risk factors can precipitate ICU-acquired delirium [15]. Despite light being the most powerful cue to maintain one’s circadian clock, alone even at high intensity it may not be sufficient to overcome the numerous disabling sensory experiences a patient undergoes while in the ICU.

To increase the sensitivity of our analysis in elucidating the effect of BLT on delirium incidence, we also conducted subpopulation modeling based on specific ICU risk factors. Each model was predefined based on significant findings from comparing baseline characteristics and clinical outcomes from patients who experienced delirium compared to those who did not. Analysis was therefore conducted for patients who had an increased SOFA score, administered sedatives, or stayed in the ICU for longer than three days. We found that even patients with a relatively low SOFA score (>3) had an observable reduction in delirium incidence among those receiving BLT. However, statistical significance could not be reached due to limitations in study population among those with documented delirium. To our knowledge, this is the first report of such an independent association in the ICU setting and should be further investigated. No differences were found in the analyses for the other subpopulations.

Our findings contrast with those of previous studies among non-critically ill patients where BLT had beneficial effects on cognitive function in geriatric and postoperative populations [16,17]. Several explanations can shed light on this discrepancy. First, many patients treated in the ICU have varying degrees of pain making the provision of adequate analgesia and sedation essential to appropriate care [18]. As sedatives can significantly distort one’s circadian rhythm, the chemical stimuli may have diminished the therapeutic effects from BLT [19]. Second, sedated patients had their eyes closed. As light best stimulates retinal input through photosensitive ganglion cells to modulate the circadian rhythm, closed eyes can dull the bright light therapeutic effect due to light attenuation by the eyelid [20]. Third, while BLT improved light exposure among critically ill patients, difficulties with adherence were noted. As the lightbox provided therapy at the bedside, patients can freely change their position in bed or adjust the bedframe angle to dull their bright light exposure. Patients who are outside the lightbox’s therapeutic range risk diluting the intended high-intensity light exposure needed to elucidate any biological effect BLT may have [21]. It is possible that unsuccessful application of the intervention accounts for the lack of difference seen between cohorts.

Although the underlying causal relationship between circadian rhythm and delirium remains unknown, the general assumption is that maintenance of the biological clock, thereby promoting normal sleep-wake cycles, can reduce the chances of developing ICU delirium. However, BLT’s beneficial effects for ICU patients have largely derived from studies primarily with non-critically ill patients [8,22]. As direct causal relation between disturbed sleep-wake cycles and delirium have not been fully substantiated, it is possible that both pathologies are a result of systemic and central nervous system inflammation commonly associated with a number of disorders including trauma and sepsis [23] The process involves the coordinated release of proinflammatory mediators and systemic cytokines during the acute phase of an illness that can dramatically alter the scope and character of brain injury. A study by Hong et al. was able to correlate the expression of inflammatory transcription factor NF-κB to the inhibition of circadian rhythm repressors leading to disrupted sleep patterns [24]. Furthermore, McNeil et al. recently demonstrated that inflammatory mediators interleukin-6 and interleukin-8 were significantly associated with prolonged delirium duration in critically ill patients [25]. These new findings may suggest that ICU patients are less susceptible to external manipulation of their circadian rhythms compared to their non-ICU counterparts.

Our study has several limitations that need to be addressed. First, this is a single-center study, which limits its external validity due to the local ICU patient population, management, and delirium prevention protocols which might differ from other ICUs. The population was largely rural Caucasian adults, and we cannot rule out whether other patient populations (other ethnic groups) or categories of patients (cardiovascular surgery or transplant surgery) may benefit from BLT. The number of patients who completed our protocol as outlined limited the pool of delirious patients in our study. One source was related to the nature of our enrollment process as a member of the research team and a patient with decision-making capacity or a guardian needed to be present to consent for study participation. Often patients admitted to the ICU can have a less predictable hospital course, which makes coordinating efforts to enroll a patient in our study within the specified 24-hour timeframe an added obstacle. Therefore, it is possible our study has missed a proportion of patients reflective of the true local ICU population.

The nature of the intervention also meant that masking of the study group was not possible. Despite the negative findings in our study, there is a possibility that BLT could have influenced the care team’s performance and the manner of care delivered to patients, which could not be ruled out in the study. We also chose to use the CAM-ICU tool to detect and diagnose delirium which also has inherent limitations. Studies have demonstrated consistent higher specificity but lower sensitivity compared to the other commonly used delirium screening tool, the Intensive Care Delirium Screening Checklist [26]. This is sobering as the CAM-ICU is capable of capturing disturbances in attention and cognition changes but may not adequately capture the fluctuating nature of these disturbances as the evaluations are done at one point in time at the discretion of the care team. Additional barriers to CAM-ICU utilization may include lack of specialized training on ICU delirium and the CAM-ICU screening tool among ICU nurses [27]. Importantly, this may lead to underdiagnosis of delirium at our institution. Nevertheless, we used the CAM-ICU at least twice per day to reduce the chance of false-negative results in ICU delirium patients.

We did not control for other precipitating risk factors native to the ICU environment that are associated with delirium such as noise and sedative protocols, but we believe these factors would be unlikely to affect our results as the cohorts were well randomized. The strengths of our study are the number of patients enrolled, the fixed BLT schedule in the intervention group, and the high adherence rate among our study participants.

## Conclusions

In this study, high-intensity BLT of shorter duration was not effective in curtailing the cumulative incidence or duration of ICU-acquired delirium compared to standard hospital lighting. Future studies should focus on expanding ease of patient enrollment and improving adherence to BLT. To better elucidate whether restoring the circadian rhythm can influence ICU delirium, future investigations should also consider a two-pronged approach focused on improving BLT during the day and minimizing light exposure at night potentially using black-out masks. Furthermore, a multifaceted intervention based on limiting unnecessary noise, light exposure, and sleep disruption could also be beneficial to minimize environmental cues for ICU patients at high risk for delirium.
